# Molecular Analysis of Short- versus Long-Term Survivors of High-Grade Serous Ovarian Carcinoma

**DOI:** 10.3390/cancers14174198

**Published:** 2022-08-30

**Authors:** Elaine Stur, Emine Bayraktar, Graziela Zibetti Dal Molin, Sherry Y. Wu, Lingegowda S. Mangala, Hui Yao, Ying Wang, Prahlad T. Ram, Sara Corvigno, Hu Chen, Han Liang, Shelley S. Tworoger, Douglas A. Levine, Susan K. Lutgendorf, Jinsong Liu, Kathleen N. Moore, Keith A. Baggerly, Beth Y. Karlan, Anil K. Sood

**Affiliations:** 1 Department of Gynecologic Oncology & Reproductive Medicine, The University of Texas MD Anderson Cancer Center, Houston, TX 77030, USA; 2 Medical Oncology Department, Beneficencia Portuguesa de Sao Paulo, Sao Paulo 13900-400, Brazil; 3 School of Biomedical Sciences, The University of Queensland, St. Lucia, QLD 4072, Australia; 4 Department of Bioinformatics, The University of Texas MD Anderson Cancer Center, Houston, TX 77030, USA; 5 Department of Systems Biology, The University of Texas MD Anderson Cancer Center, Houston, TX 77030, USA; 6 Department of Cancer Epidemiology, Moffitt Cancer Center, Tampa, FL 33612, USA; 7 Division of Gynecologic Oncology, New York University, New York, NY 11580, USA; 8 Department of Psychological & Brain Sciences, The University of Iowa, Iowa City, IA 52242, USA; 9 Department of Pathology, The University of Texas MD Anderson Cancer Center, Houston, TX 77030, USA; 10 Department of Gynecologic Oncology, The University of Oklahoma, Oklahoma City, OK 73117, USA; 11 Department of Obstetrics and Gynecology, University of California, Los Angeles, CA 90095, USA

**Keywords:** long-term survival, short-term survival, ovarian cancer, HGSC, TMEM62

## Abstract

**Simple Summary:**

Ovarian cancer is commonly associated with poor survival; patients with a diagnosis of high-grade serous ovarian carcinoma (HGSC) have an overall survival of 39% at 5 years. For reasons not well known, about 32% of ovarian cancer patients survive 10 years or more. In this study, our goal was to determine the molecular differences that drive long-term survival (LTS) in patients with HGSC. Indeed, this study shows that patients with LTS have a distinct pattern of gene expression, with TMEM62 being related to LTS. Increased TMEM62 expression led to decreased proliferation of ovarian cancer cells in vitro and decreased tumor burden in vivo.

**Abstract:**

Despite having similar histologic features, patients with high-grade serous ovarian carcinoma (HGSC) often experience highly variable outcomes. The underlying determinants for long-term survival (LTS, ≥10 years) versus short-term survival (STS, <3 years) are largely unknown. The present study sought to identify molecular predictors of LTS for women with HGSC. A cohort of 24 frozen HGSC samples was collected (12 LTS and 12 STS) and analyzed at DNA, RNA, and protein levels. OVCAR5 and OVCAR8 cell lines were used for in vitro validation studies. For in vivo studies, we injected OVCAR8 cells into the peritoneal cavity of female athymic nude mice. From RNAseq analysis, 11 genes were found to be differentially expressed between the STS and LTS groups (fold change > 2; false discovery rate < 0.01). In the subsequent validation cohort, transmembrane protein 62 (*TMEM62*) was found to be related to LTS. CIBERSORT analysis showed that T cells (follicular helper) were found at higher levels in tumors from LTS than STS groups. In vitro data using OVCAR5 and OVCAR8 cells showed decreased proliferation with *TMEM62* overexpression and positive correlation with a longevity-regulating pathway (KEGG HSA04213) at the RNA level. In vivo analysis using the OVCAR8-TMEM62-TetON model showed decreased tumor burden in mice with high- vs. low-expressing TMEM62 tumors. Our results demonstrate that restoring *TMEM62* may be a novel approach for treatment of HGSC. These findings may have implications for biomarker and intervention strategies to help improve patient outcomes

## 1. Introduction

Ovarian cancer, the most lethal gynecologic malignancy in the United States and other developed countries [1], is a heterogeneous disease with multiple histologic subtypes [2]. Among these subtypes, HGSC is the most common, with the vast majority of patients presenting with high-stage disease [2]. Patients with HGSC, despite having the same underlying histologic features, often show highly variable outcomes [3]. Despite the great impact of new therapies, survival rates have plateaued. Treatment of ovarian cancer typically includes tumor reductive surgery followed by systemic administration of platinum and taxane drugs [4]. Over the past 20 years, 5-year survival has improved substantially, but long-term survival (LTS), here defined as living 10 years or longer after diagnosis, has remained relatively low [5,6].

About half of patients with a diagnosis of advanced ovarian cancer die of disease within 5 years [7]; however, about 32% of patients with this disease survive for 10 or more years [8], although the percentage is likely lower for those with HGSC. Understanding the factors that predict LTS for this typically highly fatal disease may open up new opportunities for therapeutic interventions; however, few studies have examined predictors of LTS. Epidemiological studies of ovarian cancer suggest that diagnosis at an early age and stage as well as having low-grade and non-serous histology are predictors of LTS. Other characteristics include complete gross resection (CGR) and limited intraperitoneal spread [7]. For HGSC specifically, patients with LTS have a younger age at diagnosis, undergo optimal surgical cytoreduction, and have primary platinum-sensitive disease [9]. At a molecular level, patients with LTS commonly present with BRCA1 or BRCA2 mutations [9]. In some cases however, patients who do not present with these characteristics can become an LTS, but the underlying reasons are unclear [8,10].

Altered levels of gene expression in a tumor may reflect molecular features related to LTS versus short-term survival (STS; commonly defined as survival < 3 years) [3,11]. Previous studies have demonstrated that molecular characterization of the tumor microenvironment can identify patterns associated with patient survival [12,13]. Given the current state of poor clinical outcome for patients with HGSC, a better understanding of these differences is needed to aid in the development of novel therapeutic and more individualized strategies [14,15,16]. The goal of the present study was to identify molecular and cellular markers that differentiate LTS from STS in HGSC. Two cohorts of LTS and STS were analyzed at DNA, RNA and protein levels as well as for immune cell populations. We also investigated the role of *TMEM62*, a 643-amino acid transmembrane protein, as a possible marker of LTS.

## 2. Materials and Methods

### 2.1. Patients

For the discovery cohort, 24 frozen HGSC samples were collected at The MD Anderson Cancer Center (MDACC; Houston, TX, USA). Of these, 12 were from patients with LTS and 12 from those with STS. The patients with LTS included those who lived ≥10 years after initial diagnosis with or without recurrent disease during follow-up. For patients with STS, inclusion criteria included having at least 12 months between initial diagnosis to date of first recurrence (to exclude those with initial platinum resistance) and surviving <3 years after diagnosis. Table 1 describes the main characteristics of these patients.

A second cohort was used for validation, with 29 additional frozen HGSC samples (15 LTS and 14 STS) collected at MDACC and Cedars-Sinai Medical Center (Los Angeles, CA, USA) (Supplementary Table S1 describes the validation cohort). The samples were collected under protocols approved by the Institutional Review Board at MD Anderson and Cedars-Sinai Medical Center and all samples were collected after written informed consent was obtained from patients.

### 2.2. Cell Lines and Culture

Cell lines were obtained from the MD Anderson Characterized Cell Line Core Facility, which supplies authenticated cell lines. OVCAR5 and OVCAR8 cell lines, both derived from human high-grade serous carcinomas, were maintained in Dulbecco’s Modified Eagle medium (DMEM) and Roswell Park Memorial Institute medium (RPMI), respectively, and supplemented with 10% fetal bovine serum and 1% gentamicin. Human embryonic kidney 293 cells (293T cells) were maintained in the same conditions as OVCAR5 cells. All cells were maintained at 37 °C in 5% CO_2_. All cell lines were tested frequently for Mycoplasma by using a Universal Mycoplasma Detection Kit (ATCC) and fingerprinted by short tandem repeat (STR) analysis by the Cell Line Core.

### 2.3. Molecular Analyses

#### 2.3.1. DNA Sequencing

DNA was extracted from frozen patient tumor samples with use of TRIzol (phenol-chloroform), according to the manufacturer’s protocol. To perform whole-genome sequencing, 6 μg of tumor DNA was used and lymphocyte DNA was used as control. The sequencing coverage was 8–10× and all of the samples showed here passed quality control. The sequencing was performed with the AB 5500 Genetic Analyzer with 75bp × 35bp paired end. The analyses were performed as follows, with use of various tools for mutations and copy number variation. The BAM files were processed with use of Pindel [17] to call somatic deletions, insertions, tandem duplications, and inversions. Only calls supported by at least two reads were extracted and counted for each of the four categories per sample. The samples were also analyzed by using HMMcopy [18] to estimate the log2 ratios within consecutive 1000-bp windows along the chromosomes after correcting GC bias and normalization. Normalized log2 ratios were segmented by using circular binary segmentation [19] to detect regions of gains and losses in the tumor samples in reference to the normal sample. The cutoff used was *p*-value = 0.05 and seg.mean equal 0.4. To determine whether there were differences between LTS and STS in copy number alterations, the segmented values were analyzed with use of the cghMCR package of the Bioconductor project [20] by summarizing the gains/losses found in samples in each of the two groups based on the segmented values. To identify genes showing statistically different copy numbers in the LTS or STS groups, we used the Wilcoxon rank test. The numbers of samples analyzed varied between analyses (DNA, RNA and protein) since the amount of sample tissue was not always adequate to perform all required analyses or because samples needed to be excluded because of the low quality of biological material or poor data quality. The precise number of samples analyzed is described for each step in the results section.

#### 2.3.2. RNA Sequencing

RNA was extracted from the tumor samples by using TRIzol (phenol-chloroform), according to the manufacturer’s protocol. To perform the whole transcriptomics analysis, around 2 μg of tumor RNA was used for sequencing through SOLiD next-generation sequencing, per the protocol. RNA and miRNA were sequenced on a 5500 Series SOLiD Genetic Analysis System. Life Technologies’ LifeScope Genomic Analysis Software 2.5.1 (Carlsbad, CA, USA) was used to process the raw reads, generate BAM files, and extract raw feature counts in the initial 24 samples. Human reference build GRCh37/hg19 was used as the reference genome. The R package DeSeq2 [21] was used to normalize and analyze the read counts across all samples. Generalized negative binomial models with Wald tests were used to identify genes that were significantly differentially expressed between STS and LTS. Beta-Uniform Mixture (BUM) models [22] were used to assess false discovery rates (FDRs). mRNAs or miRNAs with estimated FDR of <0.01 and fold change of >2 between groups were selected for further validation.

#### 2.3.3. Estimates of Immune Contextures

We estimated the relative abundance of immune contextures by using a standard CIBERSORT algorithm with 500 permutation runs on the web server at https://cibersort.stanford.edu/ (accessed on 9 August 2022). We applied CIBERSORT Absolute mode to estimate the absolute immune score of each cell type in the bulk RNA sequencing of 12 LTS and 12 STS patients. Wilcoxon rank sum tests were used to test against the null hypothesis of no difference in absolute scores between STS and LTS samples.

#### 2.3.4. Reverse-Phase Protein Array (RPPA)

Protein was extracted from frozen tissue by using a lysis buffer (1% Triton X-100, 50 mM HEPES, pH 7.4, 150 mM NaCl, 1.5 mM MgCl_2_, 1 mM EGTA, 100 mM NaF, 10 mM Na pyrophosphate, 1 mM Na_3_VO_4_, 10% glycerol with protease and phosphatase inhibitors) and 4× SDS buffer (40% glycerol, 8% SDS, 0.25 M Tris.HCL, pH 6.8 and beta-mercaptoethanol at 1/10 of volume). Wilcoxon rank sum tests were used to identify the differentially expressed proteins between STS and LTS. BUM models were used to adjust for the multiple comparisons. Differences in protein expression with FDRs of <0.1 and fold changes of >1.5 were considered to be statistically significant between groups.

### 2.4. Plasmid Constructs and Delivery

The human TMEM62 plasmid (pLenti-GIII-CMV-RFP-2A-Puro-ABM #LV482513) (abm, Richmond, BC, Canada) was used for transient transfection, and the same plasmid was modified to a TetOn system to create the stable cell line (OVCAR8-TMEM-TetON). For virus production, 293T cells were grown for 1 week in DMEM (10% fetal bovine serum) antibiotic-free, before virus production. Cells were seeded at 3.8 × 10^5^ cells/mL in 10-cm dishes and incubated overnight. To generate lentivirus particles, 293T cells were transfected by using Lenti-X™ Packaging Single Shots (VSV-G), according to the manufacturer’s protocol. Transfection medium was incubated for 48 h, after which virus particles were collected and concentrated with use of a Lenti-X™ Concentrator (Takara Bio, San Jose, CA, USA).

For cell infection, 1 × 10^5^ cells were infected with 0.2 mL of virus combined with polybrene at a final concentration of 10 μg/mL, in 0.2 mL of medium. The initial medium was replaced with complete medium after 16 h. Antibiotic selection was started after 48 h of incubation in complete medium. The cells were treated with 2 µg/mL of puromycin for 48 h twice, then sorted by flow cytometry. The cells were then expanded, frozen, and used for the following experiments.

### 2.5. Transient Overexpression

OVCAR5 and OVCAR8 cells were transfected with either the control or the TMEM62 plasmid by using 1 μg per well in a 6-well plate with 3 μL of FuGENE HD Transfection Reagent (Promega, Madison, WI, USA) according to the manufacturer’s protocol. The cells were used for further experiments after 48 or 72 h of initial transfection.

### 2.6. cDNA Synthesis and Quantitative Real-Time Reverse-Transcriptase Polymerase Chain Reaction

For target validation in the second cohort, 0.5 μg of total RNA was transcribed into cDNA by using a Verso cDNA synthesis kit (Thermo Scientific, Pittsburgh, PA, USA). For targeted studies, the TMEM62 primers that were used were All-in-One qPCR primer (GeneCopoeia, Rockville, MD, USA, cat# MQP041184). The reaction was performed according to the manufacturer’s protocol, with a 20-μL total volume that contained 2.0 μL of cDNA, 2 μL of All-in-One primer, and 10 μL of Sybr Green Master Mix (Applied Biosystems, Foster City, CA, USA). Quantitative real-time PCR (qRT-PCR) was performed in triplicate by using the Applied Biosystems 7500 series with initial denaturation at 95 °C for 15 min, followed by 40 cycles of 95 °C for 15 s and 60 °C for 1 min. The relative expression of target gene mRNA was normalized to the amount of 18S in each sample by using the ΔΔCt method.

### 2.7. Apoptosis, Proliferation, and Cell Cycle Assay

The effects of apoptosis, proliferation, and the cell cycle were analyzed after transient overexpression of TMEM62 for 48 and 72 h in OVCAR5 and OVCAR8 cell lines, as described above. The relative percentage of apoptotic cells was assessed by using an Annexin V-Coupled Fluorescein Isothiocyanate (FITC) Apoptosis Detection Kit (BD Pharmingen), according to the manufacturer’s protocol, and DAPI (1 µg/mL) was added for cell cycle analysis. For proliferation analysis, a Click-iT™ Plus EdU Alexa Fluor™ 488 Flow Cytometry Assay Kit was used according to the manufacturer’s protocol.

### 2.8. Immunohistochemistry Analysis

Human samples: Matched paraffin-embedded ovarian cancer tissue samples were obtained from the frozen LTS and STS samples to evaluate the CD8 count (n = 10 per group) of the discovery cohort. Tissue sections were deparaffinized and dehydrated, and heat-induced epitope retrieval (pH 9.0) was used; cells were then blocked in 3% fish gelatin. After blocking, sections were incubated with a monoclonal anti-CD8a antibody (1:100; DAKO, catalog no. m710301-2) at 4 °C overnight. The next day, the sections were washed and incubated with horseradish peroxidase-conjugated rat anti-mouse IgG2a (1:200; Jackson ImmunoResearch Laboratories, West Grove, PA, USA) for 1 h at room temperature. Sections were stained with DAB and counterstained with hematoxylin. The images were obtained with use of a Leica camera (Wetzlar, Germany) at 400× magnification. Positive cells were counted with use of Image J.

In vivo (mouse) samples: TMEM62 expression (TMEM62, 1:50, Thermo Fisher Scientific, Waltham, MA, USA, #PA5-60719) was performed as described above. For apoptosis, we used a Click-iT TUNEL colorimetric IHC detection kit (Thermo Fisher Scientific, #C10625) according to the manufacturer’s protocol. The images were obtained with use of a Vectra Polaris Imaging System (Akoya Biosciences, Marlborough, MA, USA) at 40× magnification. Cells were counted by using pathology image analysis software (Visiopharm, Hørsholm, Denmark). Human samples were quantified for TMEM62 using this same condition.

### 2.9. In Vivo Experiments

We purchased 6- to 12-week-old female athymic nude mice (strain NCRNU/RRID: IMSR_TAC: ncrnu) from Taconic Biosciences (Rensselaer, NY, USA). Five animals were kept in each cage and maintained under specific pathogen-free conditions. The in vivo study was performed in accordance with the American Association for Laboratory Animal Care institutional guidelines. The study protocol was approved and supervised by the Institutional Animal Care and Use Committee (IACUC) at MD Anderson.

To analyze the effect of overexpressing TMEM62 in an in vivo model, we injected 4 × 10^6^ OVCAR8-TMEM62-TetON cells intraperitoneally (n = 15 per group) in 0.2 mL of Hank’s balanced saline solution. Mice were divided into 3 groups: control; standard diet; group 1 (G1): animals started to receive doxycycline-diet (200 mg/kg) on the day of cell injection; and group 2 (G2): animals started to receive doxycycline diet on day 14 after cell injection. Both groups received the doxycycline diet until the end of the study. For assessing tumor growth, the animals were observed for 65 days. For the survival experiment, they were kept until they became moribund. Tumor uptake was inferred by palpation. For takedown, mice were euthanized by CO_2_ exposure followed by cervical dislocation when they became moribund in any group due to disease. For data acquisition, all the observable nodules were collected and quantified by number of nodules and total weight of nodules. The group assignment was blinded for individuals performing the procedures. Tissue specimens were either fixed by using 10% buffered formalin, frozen in OCT (Miles, Inc., Elkhart, IN) or were snap-frozen in liquid nitrogen.

### 2.10. Statistical Analyses

All in vitro experiments were done at least in technical triplicates with differences by group assessed by the Student’s *t* test or ANOVA (for comparison of all groups). For in vivo experiments, tumor weight was analyzed by one-way analysis of variance, and groups were compared by ANOVA. GraphPad Prism version 8.0 was used for all analyses. All data are presented as mean ± SD and two-sided unless otherwise indicated. Differences were considered statistically significant if *p* value was <0.05 according to a two-tailed test.

## 3. Results

### 3.1. Omics Analysis

To understand potential molecular differences between LTS and STS, we identified a cohort of 24 patients with HGSC, stages IIIC or IV (12 LTS and 12 STS). Both groups had an average age of 61 years.

The molecular data generated from DNA sequencing included analyses of mutational data from six LTS and four STS samples. Due to low coverage, we kept the samples with enough depth for variant calls. Furthermore, since HGSC is a copy number-driven disease [23], we analyzed copy number variation from 11 LTS and 12 STS samples. The mutation data included 553 mutations recorded in the Single Nucleotide Polymorphism database (dbSNP) and 8878 novel mutations. Only two genes (ROCK1P1 and NGLY1) had *p* values < 0.05 for differential counts between groups. Both genes had mutations in three of four ST survivors (75%) but showed no mutations in the six LT survivors. Additionally, MT-CYB was mutated in three of four STS but was not mutated in any of the LTS samples (*p* = 0.033). A complete list of mutations differentially found between these groups and filtered by gnomAD is shown in Supplementary Table S2. We do recognize that the low coverage of DNA sequencing may have hindered the identification of all the mutations between these groups.

The copy number segmentation data showed that the profiles of these two groups were not substantially different (Supplementary Figure S1A). Supplementary Figure S1B shows a relative count between both groups and Supplementary Figure S1C shows a copy number summary representing the segment of gain or loss (SGOL) score across the genome for both groups. STS group showed a tendency to have more copy number gains in 2q and 3q and copy number losses in 7p, 8p, and 15q, but these changes were not statistically significant (*p* > 0.05; (Supplementary Table S3)). Similar results were observed for the RPPA analysis, with no statistically significant differences between groups; however, a few proteins (e.g., PAR, Claudin-7, RB) had higher levels in the STS group than in the LTS group (Supplementary Figure S1D,E). All the proteins analyzed are included in Supplementary Table S4.

**Figure 1:**
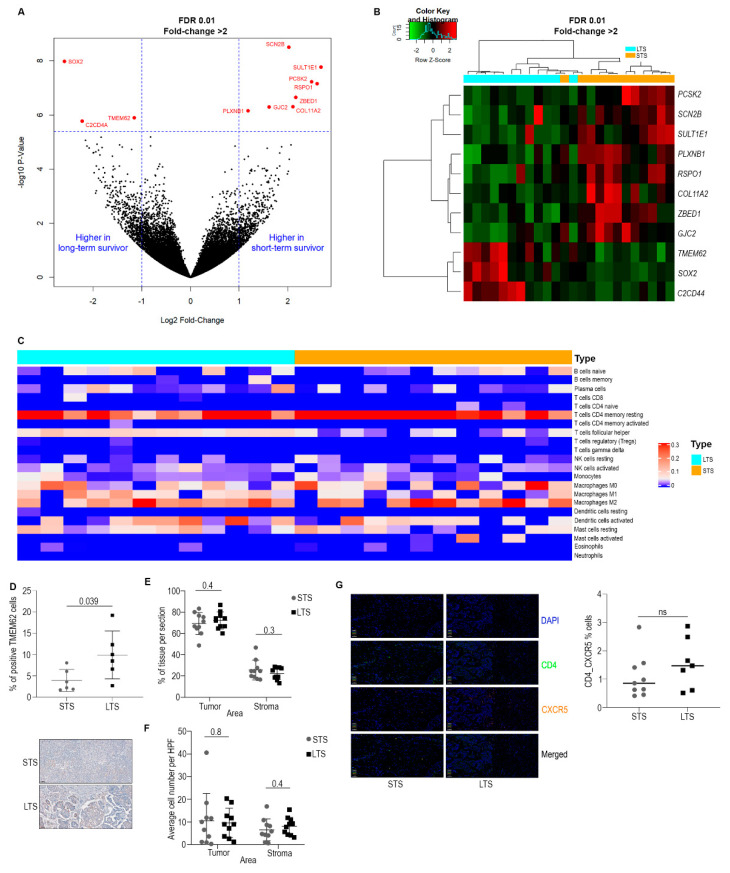
Molecular analysis of high-grade serous ovarian carcinoma (HGSC) samples from patients with long-term survival (LTS) or short-term survival (STS). (**A**) Volcano plot from RNASeq data showing the genes with highest expression in both groups. (**B**) Heat map showing the expression levels of genes highly expressed in LTS. (**C**) CIBERSORT analysis from patients with LTS or STS. (**D**) IHC validation of higher expression of TMEM62 in the LTS group. Top: IHC quantification using Visiopharm. Bottom: Representative images of LTS and STS groups stained with TMEM62 antibody. Scale: 50 µm (**E**) Percentages of tissue area (tumor and stroma). (**F**) Number of infiltrating CD8 cells per tissue area. (**G**) Percentage of positive T_FH_ per group. Right: OPAL staining using CD4 and CXCR5 antibodies. Left: Percentage of positive double stained cells in the whole tissue area.

Next, we examined gene expression differences by using SOLiD next-generation sequencing. Supplementary Figure S2A shows a PCA generated from RNA sequencing analysis from the LTS and STS samples. The analysis showed a total of 11 genes identified as differentially expressed between STS (n = 12) and LTS (n = 12) patients (RNAseq, fold change > 2, *p* < 1 × 10^−5^, FDR < 0.01) (Figure 1A,B). A secondary screen was used to eliminate genes expressed below threshold levels. Eight genes were found to be highly expressed in samples from patients with STS in the initial RNA sequencing analysis. Of these, five had sufficient expression for potential knockdown (PCSK2, SCN2B, SULT1E1, PLXNB1, and RSPO1). Three genes were overexpressed in LTS versus STS groups, with TMEM62 having the highest expression in patients with LTS. In the subsequent validation cohort, TMEM62 had the most significant result of the initial genes of interest, with qRT-PCR data demonstrating an average of 3-fold higher expression of TMEM62 mRNA in LTS (n = 15, Supplementary Figure S2B) compared with STS (n = 14) groups. Due to this gene being observed to have the highest expression in two different cohorts, we decided to pursue TMEM62 for further analyses. The higher expression of TMEM62 in the LTS groups was also validated by IHC, showing a statistically significant increase at the protein level (Figure 1D).

To explore transcriptomic differences between LTS and STS, we used Ingenuity Pathway Analysis (IPA). Using an FDR of 0.2, we observed downregulation of the integrin signaling and GP6 pathways and upregulation of TH1, natural killer cell signaling, and crosstalk between dendritic cell and natural killer cell pathways in LTS versus STS samples (Supplementary Figure S2C). Other altered pathways that met our significance threshold included upregulation of cytotoxic T lymphocyte–mediated apoptosis of target cells and communication between innate and adaptative immune cells. Additionally, upstream analysis showed that STAT1 and many other immune-related upstream regulators (e.g., IFNa, IFNγ, IL21, IL27, IL10—complete upstream regulators are shown in Supplementary Table S5) were predicted to be activated in the LTS group. Together, these transcriptomics data indicated that LTS samples had increased activation of the innate immune response, recruitment of T lymphocytes, and cellular homeostasis (Supplementary Figure S1).

### 3.2. Characterization of Immune Cell Populations

To understand the role of the tumor microenvironment, immune cell populations were characterized through deconvolution of mRNA sequence data (CIBERSORT), which identified 22 cell populations. Results showed that the largest difference was for T follicular helper cells (T_FH_) in which LTS samples had higher scores than did STS samples (*p* = 0.0007; Figure 1C), indicating higher infiltration of these cells in the LTS samples. Due to the well-known role of CD8 cells, we also quantified CD8 infiltration in the tumor and stroma, and concordant with CIBERSORT analysis, we did not observe significant differences between the two groups overall or within stroma and tumor areas (Figure 1E,F). We also quantified T_FH_ cells, and we observed a trend of increased infiltration of these cells in the LTS group. Concordant with CIBERSORT, not all STS tumors show low infiltration, but there is a clear trend indicating that T_FH_ cells are more frequently found in the LTS samples (Figure 1G).

### 3.3. Effects of Changes in TMEM62 Expression

**Figure 2:**
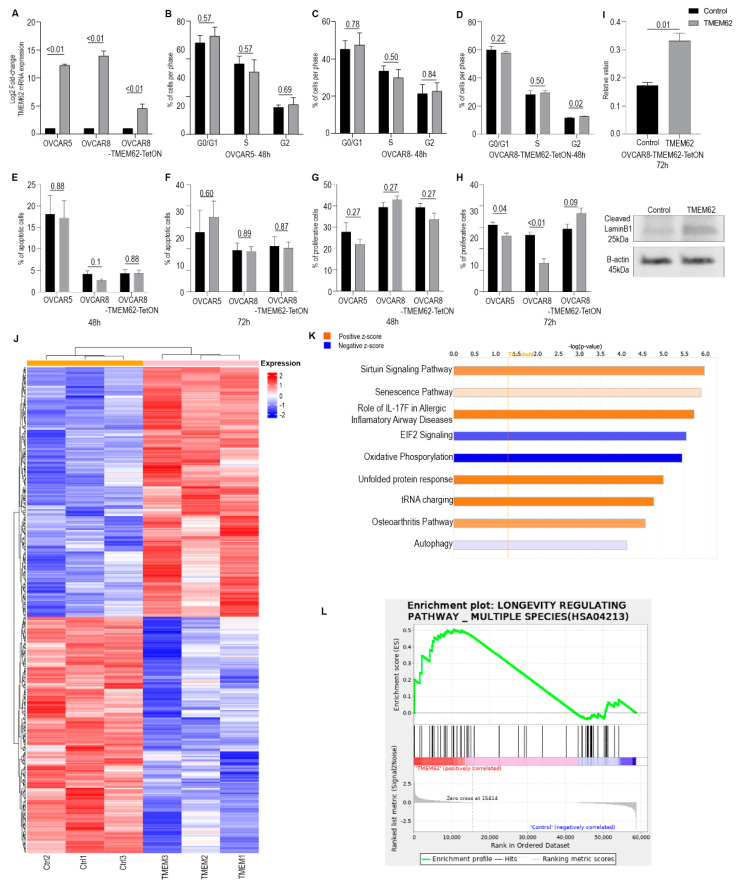
In vitro data for OVCAR5 and OVCAR8 cells with transient overexpression of TMEM62. (**A**) qRT-PCR results from TMEM62 overexpression using a transient method and a dox-inducible model. Functional analysis includes (**B**) cell cycle of OVCAR5 cells, (**C**) OVCAR8, and (**D**) OVCAR8-TMEM62-TetON cells at 72 h; apoptosis assay at (**E**) 48 h, and (**F**) 72 h using OVCAR5, OVCAR8, and OVCAR8-TMEM62-TetON cells, and proliferation assay of the same cells overexpressing TMEM62 at (**G**) 48 h and (**H**) 72 h. (**I**) Senescence measurement of OVCAR8-TMEM62-TetON. Up: Western blot quantification/ Bottom: Western blot image of Cleaved LaminB1 and β-Actin. (**J**) Heatmap of OVCAR8 cells expressing TMEM62. (**K**) Pathway analysis generated from RNASeq using IPA. (**L**) Enrichment plot from RNASeq using KEGG.

Given the correlation of TMEM62 with LTS in patients with HGSC, we next examined the functional effects of TMEM62 in preclinical models. We assessed expression levels of TMEM62 in HGSC cell lines: OVCAR5 and OVCAR8 cells showed relatively low expression of TMEM62. Using a TMEM62 plasmid, there was a 15-fold increase in TMEM62 expression with transient transfection (Figure 2A). Initially, we tried to create a stable cell line with this plasmid, but the cells did not survive. Therefore, we used transient expression; transient overexpression of TMEM62 led to decreased proliferation in short-term experiments (48–72 h, with statistically significant differences only at 72 h). TMEM62 overexpression did not affect cell cycle or apoptosis (Figure 2B–H). We also checked for senescence markers and there was a significant increase in cleaved LaminB1 levels, indicating an increase in senescence of cells overexpressing TMEM62 (Figure 2I).

We further performed RNA sequencing of OVCAR8 cells overexpressing TMEM62 at 48 h after transfection. The data showed 411 differentially expressed genes, with 211 being upregulated (*p*-adjusted < 0.05) and 200 being downregulated (Figure 2J). Using enrichment analysis with IPA, we observed an increase in multiple pathways associated with survival, such as sirtuin signaling and senescence pathways, and a decrease in EIF2 signaling (stress-related) and oxidative phosphorylation (Figure 2K). With use of KEGG, we found that the longevity-regulating pathway (KEGG HSA04213) was positively correlated with TMEM62 overexpression (Figure 2L). Multiple pathways were commonly found between IPA and KEGG analysis including an increase in cytokines (IL superfamily members) and inflammatory pathways, particularly related to innate immunity (attraction of leukocytes). Of interest, immune-related pathways were also identified in the samples from patients with LTS. We also observed that both databases showed an increase in cellular senescence with TMEM62 expression. Together, these data suggest that TMEM62 leads to changes in cell survival pathways, which could explain the increased survival of patients with increased TMEM62 expression.

### 3.4. In Vivo Studies

To determine whether the overexpression of TMEM62 affects tumor growth and progression, we developed a TetOn system cell line model using OVCAR8 cells. In vitro, treatment of OVCAR8-TMEM62-TetON cells with 0.2 µg of doxycycline for 24 h induced a 5-fold increase in TMEM62 expression (Figure 2A).

**Figure 3:**
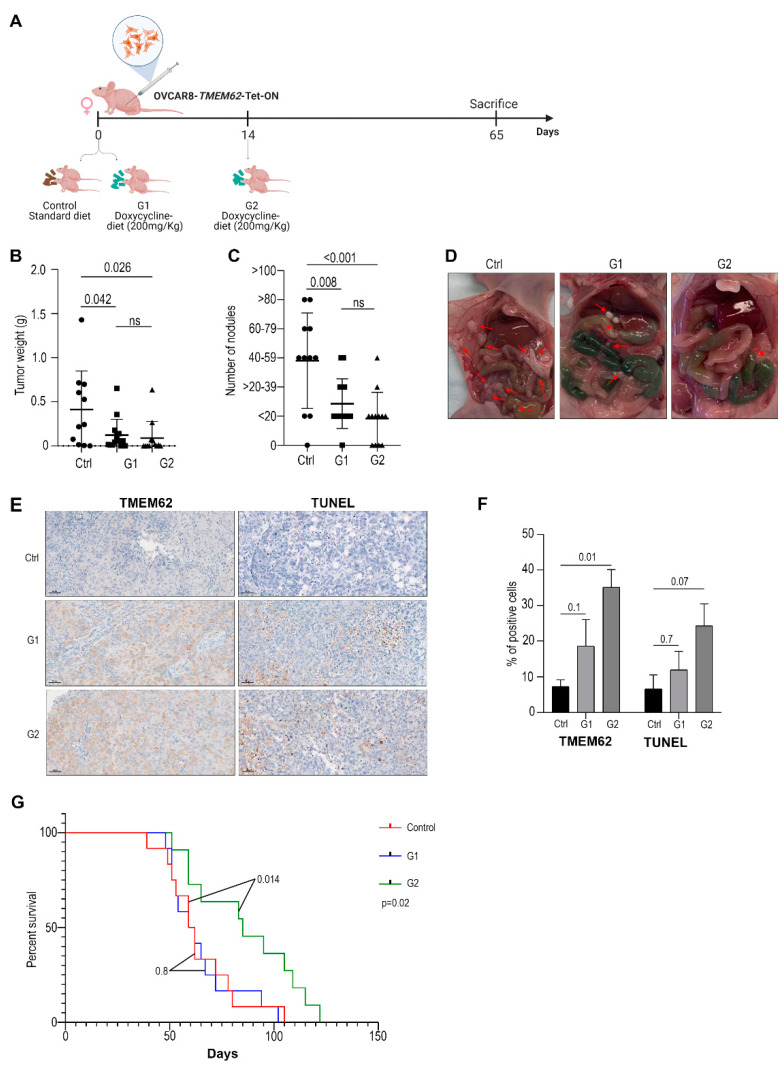
In vivo data generated by injecting OVCAR8-*TMEM62*-TetOn cells. (**A**) Representative schema of cell injection and groups included in the study. (**B**) Tumor weight and (**C**) number of tumor nodules. (**D**) Representative images of tumors in each group. Red arrows indicate the location of tumor nodules. (**E**) Representative images of TMEM62 expression (left panel) and TUNEL assay (right panel). Scale: 50 µm (**F**) Quantification of TMEM62 and TUNEL assay. (**G**) Survival experiment in OVCAR8-*TMEM62*-TetOn model.

To determine whether TMEM62 overexpression was important in early tumor establishment and/or during tumor progression, we divided the experiments into control, G1, and G2 groups (with the G1 group receiving dox-chow diet from the day of cancer cell injection and the G2 group receiving a special diet starting 14 days after cell injection; Figure 3A). The results showed a difference between the treated groups and the controls; treated groups had decreased tumor weight (Figure 3B) and the number of nodules (Figure 3C) compared with the control (Figure 3D shows a representative image of the tumors in each group), without difference in body weight (Supplementary Figure S3A). When comparing G1 and G2, we observed smaller tumor weights and fewer nodules in G2 than in G1, but this difference was not statistically significant (Figure 3C). Some outliers were identified by a Grubbs test and are highlighted in red in Supplementary Figure S3B,C. To assess TMEM62 expression in vivo, we observed an increase in TMEM62 expression in G1 (*p* = 0.1) and G2 (*p* = 0.01) compared with the control (Figure 3E,F). Additionally, we used the TUNEL assay to measure the levels of apoptosis; there was a non-statistically significant increase in positive cells in the G1 (*p* = 0.7) and G2 (*p* = 0.07) groups (Figure 3E,F).

To test if TMEM62 overexpression could increase survival, we employed the same experimental design as shown above (OVCAR8-TMEM62-TetON), and we observed a significant increase in survival of G2 group (animals started on special diet 14 days after tumor cell injection), when compared with control and G1 (Figure 3G); no significant difference was observed between control and G1. These results indicate that TMEM62 has the potential to increase survival in mouse models of high-grade serous ovarian carcinoma.

## 4. Discussion

In this multi-omics analysis of tumors comparing patients with HGSC who survived at least 10 years (LTS) versus those who survived 3 years (STS), we identified *TMEM62* as a possible target for enhancing survival. Overexpression of *TMEM62* was a feature of LTS but not of STS tumors, and increased expression led to changes in cell survival pathways, including increase in senescence markers. Senescence has been associated with a tumor suppressive process, inhibiting tumor growth, decreasing proliferation and even increase the chance of immune related events for tumor clearance [24,25,26], which may explain the mechanism by which TMEM62 could affect long-term survival. Besides that, we observed a trend for G2 arrest, which could launch the senescence program [27]. An increase in the sirtuin pathway was also observed, but the roles of this pathway in cancer pathogenesis are not fully understood [28,29]. Furthermore, the omics data indicated that LTS samples had increased activation of the innate immune response, recruitment of T lymphocytes (particularly T_FH_ cells), and cellular homeostasis.

Demographic studies have shown that ovarian cancer patients with LTS have decreased levels of CA125 [4], are younger at diagnosis [8,30], and have no residual disease after debulking surgery [31], but few studies have explored the molecular features of this group with unexpected survival trajectories. Of interest, our genomics analysis did not identify a mutational pattern that significantly differed between the groups. Yang et al. (2018) also analyzed the mutational pattern of HGSC tumors from LTS patients [31] and observed increased somatic mutational burden in the LTS group, which was not observed in our study. This difference could be related to their higher coverage in DNA sequencing as well a larger sample size.

One prior study examined differences in gene expression patterns related to LTS and found that the *MAL* gene was the most upregulated in STS patients. Tumors from LTS patients had a similar pattern of gene expression to that of tumors from patients with early-stage disease [4], suggesting that the molecular pattern of gene expression related to LTS may appear in early stages of tumorigenesis. Although our study did not identify these genes specifically or compare their patterns of expression to those of genes associated with early-stage disease, we did observe downregulation in integrin signaling and GP6 pathways in LTS, which are potentially associated with improved survival. Integrin signaling has been described as a major pathway for cell survival, adhesion, proliferation, cell cycle regulation, angiogenesis, and resistance to therapy [32,33], and drugs have already been developed targeting several integrins [34,35]. In addition, the GP6 pathway is known to be a requisite for formation of platelet aggregation, which in turn plays a role in cancer development, progression, and metastasis [36,37].

The immune profiling of the tumor microenvironment from LTS patients has not been well described. Yang et al. (2018) used RNA sequencing to predict the immune milieu and observed enrichment of the immune reactive HGSC subtype [31] in LTS but not in STS tumors, with high immune infiltration of active CD8+ cells and both activated and memory CD4+ cells. By using deconvolution techniques, we identified strong enrichment for T_FH_ cells, which are CD4+ cells, in the LTS group. T_FH_ cells assist B cells with germinal center formation, maturation, and development of high-affinity antibodies and memory B cells [38]. T_FH_ cells in cancer correlate with increased survival in melanoma [39] and in breast cancer, with extensive immune cell infiltration and increased interferon γ, CD8+ cells, and B cells [40,41]; some studies, however, suggest that T_FH_ cells may increase TGF-β and IL-10 as well as PD1+/TIM3+ cells, which could be indicative of dysfunctional CD8+ cells [42,43]. Interestingly, recent evidence supports the role of T-cell and B-cell clusters, called tertiary lymphoid structures, in improved survival in ovarian cancer patients [44,45,46], suggesting a potential role for this joint immune response in LTS. It is not clear how this process could be associated with LTS or with the mechanism by which *TMEM62* could lead to LTS; further analysis is needed.

A key finding of our study is that *TMEM62* is an indicator of LTS. Relatively little has been published about the characteristics, function, and relevance of *TMEM62*, a 643-amino-acid transmembrane protein [47], or other members of this large family of transmembrane proteins. At a subcellular level, *TMEM62* has been detected in the nucleoplasm, nucleoli fibrillar center, and nuclear and cytoplasmatic bodies [48]. Members of this family can act as both oncogenes, such as *TMEM45A* and *TMEM205*, or tumor suppressors, such as *TMEM25* and *TMEM7* [49]. *TMEM62* is expressed in all human tissues represented in The Human Protein Atlas, with high RNA expression and low protein expression in the female tissues [48]. On the basis of molecular function ontology, *TMEM62* has been classified as having hydrolase activity and involvement in cellular metabolic processing, in addition to being an integral membrane component [50].

*TMEM62* has also been shown to be expressed in the placental decidua, with a putative lipoxygenase function. *TMEM62* shares tertiary structural similarities with known human lipoxygenases, with a membrane-binding N-terminal β-barrel domain and a catalytic domain composed primarily of α-helices. Direct interactions between *TMEM62* and leukotriene A4 hydrolase have also been noted [51]. Leukotriene A4 hydrolase is an enzyme that functions to convert leukotriene A4 to leukotriene B4 (LB4). LB4 is a proinflammatory mediator that functions to prolong tissue inflammation by the recruitment and activation of granulocytes and production of cytokines [52]. In addition to its main proinflammatory effects mediated by LB4 receptor 1, LB4 also binds with lower affinity to LB4 receptor 2 (BLT2), which has been implicated in human cancer growth and proliferation. In HGSC specifically, BLT2 has been shown to promote invasion and metastasis through activation of STAT3 and upregulation of metalloproteinase 2 (MMP2) [53].

The strengths of this study include the multi-omics approach, with validation in an independent sample set as well as by orthogonal methods. However, the relatively small sample size may have limited the identification of more robust differences between tumors from patients with LTS compared with those with STS. The low coverage for DNA sequencing also may have precluded identification of differences between these populations.

## 5. Conclusions

Our study suggests that gene-level and immunological differences exist between HGSC patients who experience LTS versus STS. Our results demonstrate that restoring *TMEM62* may be a novel approach for treatment of HGSC. These findings may have implications for biomarker and intervention strategies to help improve patient outcomes.

## Figures and Tables

**Table 1:** Characterization of patients with ovarian cancer who had LTS or STS. * Samples used for whole-genome sequencing.

Sample ID	Sample Type	Age	Site	Stage
1	LT	63	Ovary	IIIC
2 *	LT	63	Ovary	IIIC
3	LT	53	Ovary	IIIC
4	LT	71	Ovary	IIIC
5	LT	49	Ovary	IIIC
6	LT	54	Ovary	IIIC
7 *	LT	67	Ovary	IIIC
8 *	LT	66	Peritoneum	IV
9 *	LT	56	Ovary	IIIC
10 *	LT	58	Ovary	IIIC
11	LT	69	Ovary	IIIC
12 *	LT	63	Ovary	IIIC
13	ST	62	Tube	IIIC
14	ST	50	Ovary	IIIC
15	ST	59	Ovary	IIIC
16	ST	57	Tube	IV
17 *	ST	64	Ovary	IIIC
18 *	ST	74	Peritoneum	IIIC
19	ST	54	Tube	IV
20 *	ST	78	Ovary	IV
21 *	ST	60	Tube	IIIC
22	ST	59	Tube	IIIC
23	ST	53	Ovary	IIIC
24	ST	66	Ovary	IIIC

## Data Availability

The molecular data generated (DNA and RNA sequencing) is deposited at dbGaP. Study Accession: phs003020.v1.p1.

## Supplementary Materials

The following supporting information can be downloaded at: https://www.mdpi.com/article/10.3390/cancers14174198/s1, Figure S1: (A) Principal component analysis (PCA) generated from copy number analysis from long-term survival (LTS) and short-term survival (STS) samples. (B) Bar plot of counts comparing LTS and STS groups. (C) Copy number summary representing the segment of gain or loss (SGOL) score across the genome for both groups. (D) Volcano plot generated from RPPA data shows no clear distinction between LTS and STS. (E) Heat map compares the levels of proteins identified by RPPA in both groups. Figure S2: (A) PCA generated from RNA sequencing analysis from long-term survival (LTS) and short-term survival (STS) samples. (B) qRT-PCR validation of genes identified in the RNASeq analysis from a second cohort of LTS and STS patients. (C) Pathway analysis generated from RNASeq using IPA using the human dataset, with absolute value z-score greater than 1.5. Figure S3: In vivo data generated by injecting OVCAR8-TMEM62-TetOn cells including the outliers identified with use of the Grubbs test. (A) Body weight, (B) tumor weight, and (C) number of tumor nodules (animal in red in the control group is an outlier for tumor weight, but not for number of nodules. G1 and G2 have the same animal as outliers for tumor weight and number of nodules. Red dots indicate possible outliers based on the Grubbs test done with the tumor weight data. (D) Raw western blot data from Figure 2I, showing the cleaved LaminB1 with merged marker (top) and βactin (bottom). Table S1: Validation cohort for RNA sequencing. Table S2: A complete list of mutations found between for LTS and STS. The gnomAD version v2.1.1 exome database and version v3.1.2 genome database was used separately to extract the allele frequencies (AF). A total AF and a non_cancer_AF were provided. Table S3: Copy number variation of LTs and STS patients. Table S4: RPPA analysis including all proteins analyzed. Table S5: Upstream regulators generated from the RNA sequencing data from LTS and STS patients.
