# Supplementary figures and images for: Molecular Analysis of Short- versus Long-Term Survivors of High-Grade Serous Ovarian Carcinoma
Source: Cancers (Basel). 2022 Aug 30;14(17):4198. doi: 10.3390/cancers14174198 (PMC9454595; doi:10.3390/cancers14174198)

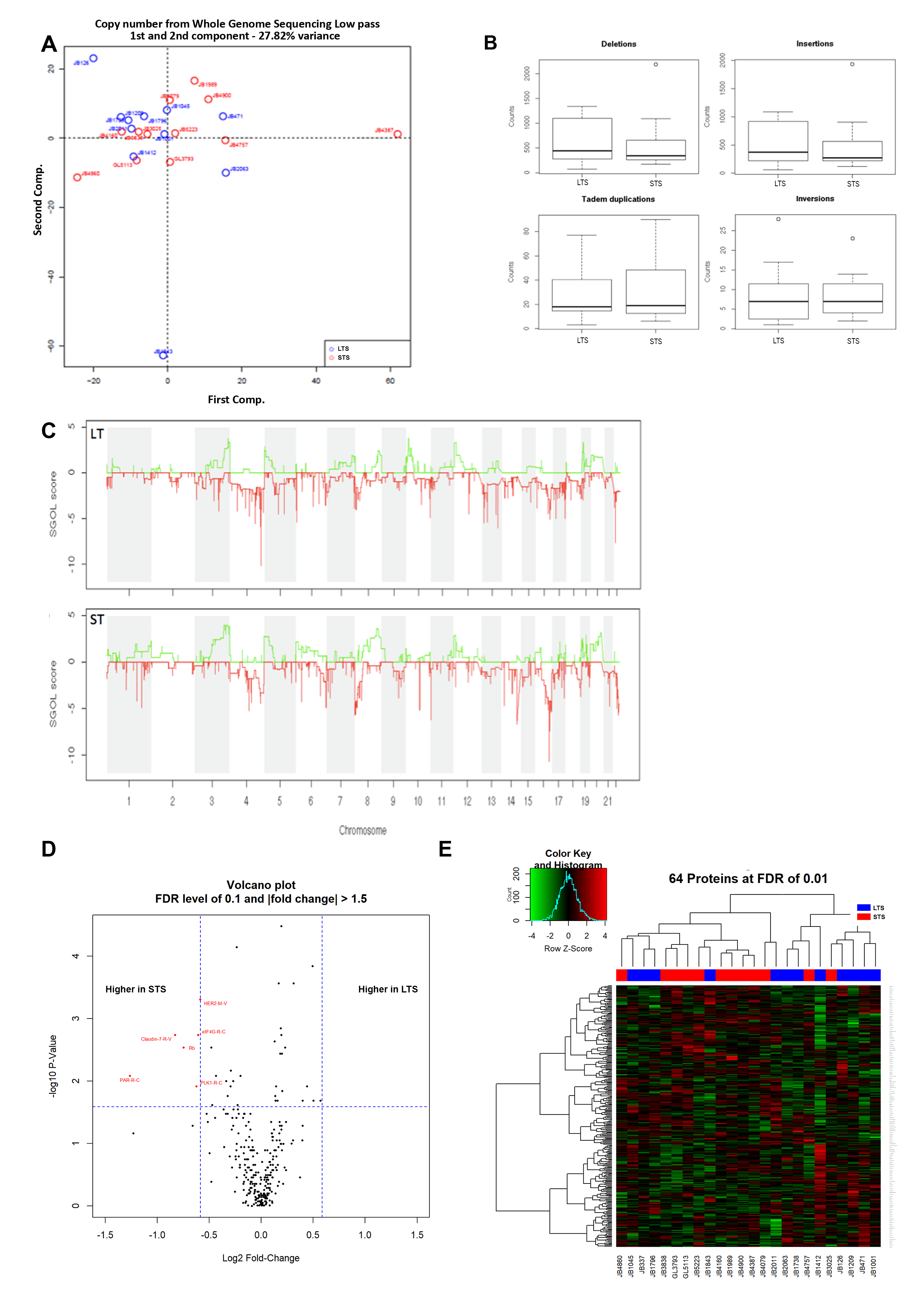

Supplement: Supplementary file 1 [file cancers-14-04198-s001.zip › cancers-1818283-supplementary/Sup Figure S1 0722222.tif]

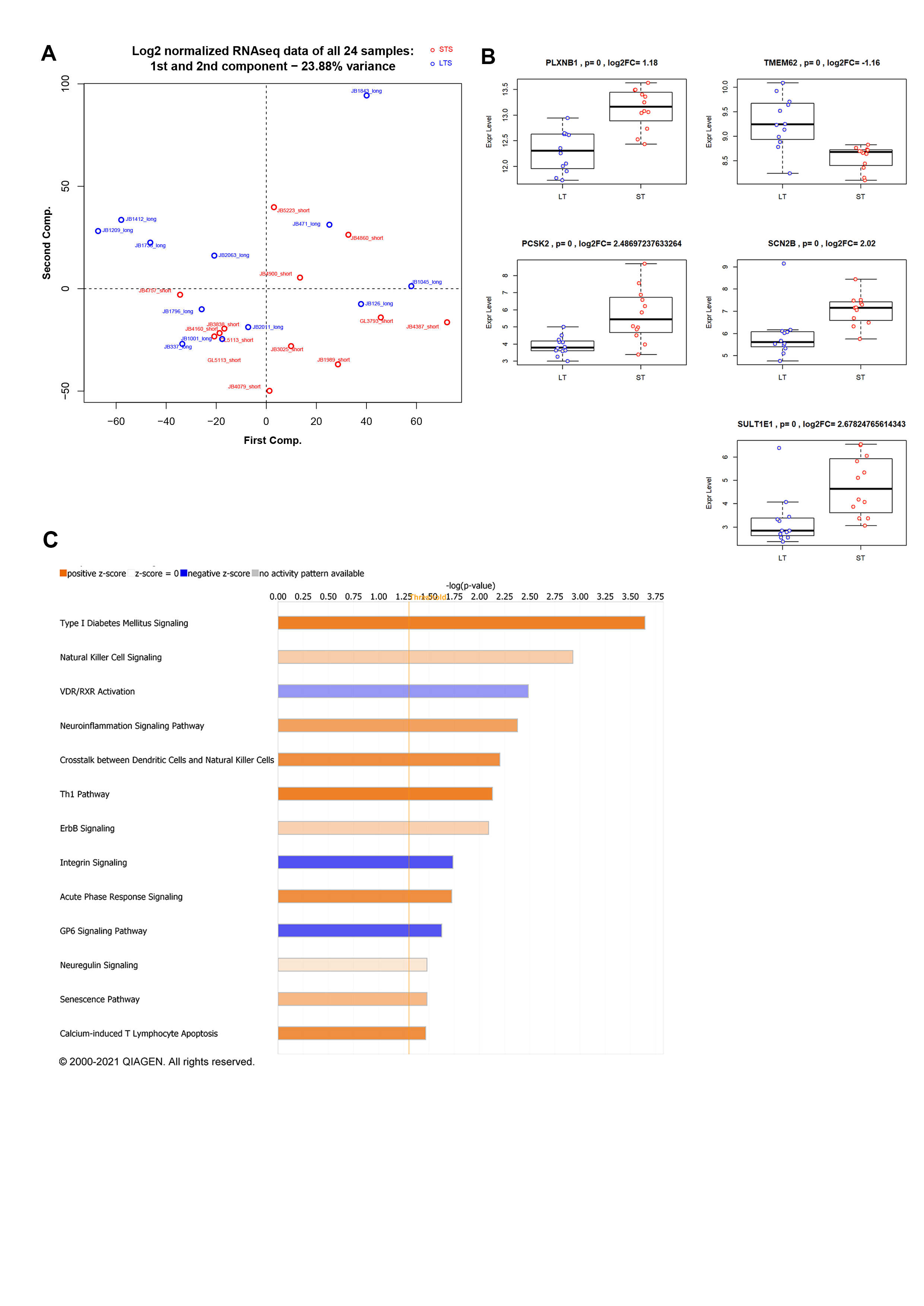

Supplement: Supplementary file 1 [file cancers-14-04198-s001.zip › cancers-1818283-supplementary/Sup Figure S2.tif]

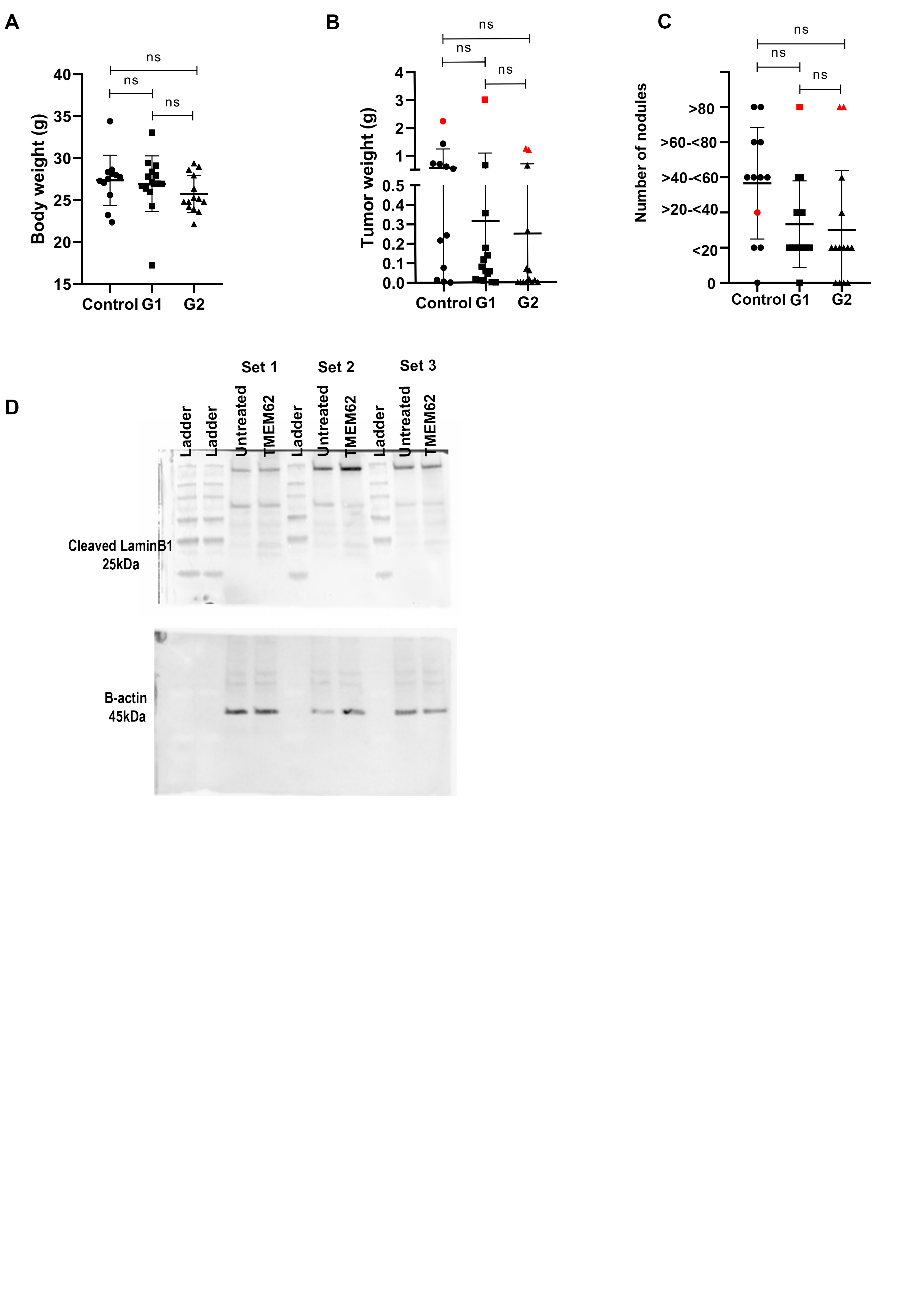

Supplement: Supplementary file 1 [file cancers-14-04198-s001.zip › cancers-1818283-supplementary/Sup Figure S3.tif]
